# Fully Automated Myocardial Strain Estimation from Cardiovascular MRI–tagged Images Using a Deep Learning Framework in the UK Biobank

**DOI:** 10.1148/ryct.2020190032

**Published:** 2020-02-27

**Authors:** Edward Ferdian, Avan Suinesiaputra, Kenneth Fung, Nay Aung, Elena Lukaschuk, Ahmet Barutcu, Edd Maclean, Jose Paiva, Stefan K. Piechnik, Stefan Neubauer, Steffen E. Petersen, Alistair A. Young

**Affiliations:** From the Department of Anatomy and Medical Imaging, University of Auckland, Auckland, New Zealand (E.F., A.S., A.A.Y.); William Harvey Research Institute, NIHR Barts Biomedical Research Centre, Queen Mary University of London, Charterhouse Square, London, England (K.F., N.A., E.M., J.P., S.E.P.); and Oxford NIHR Biomedical Research Centre, Division of Cardiovascular Medicine, Radcliffe Department of Medicine, University of Oxford, Oxford, England (E.L., A.B., S.K.P., S.N.); Department of Biomedical Engineering, King’s College London, 5th Floor Becket House, 1 Lambeth Palace Rd, London SE1 7EU, England (A.A.Y.).

## Abstract

**Purpose:**

To demonstrate the feasibility and performance of a fully automated deep learning framework to estimate myocardial strain from short-axis cardiac MRI–tagged images.

**Materials and Methods:**

In this retrospective cross-sectional study, 4508 cases from the U.K. Biobank were split randomly into 3244 training cases, 812 validation cases, and 452 test cases. Ground truth myocardial landmarks were defined and tracked by manual initialization and correction of deformable image registration using previously validated software with five readers. The fully automatic framework consisted of *(a)* a convolutional neural network (CNN) for localization and *(b)* a combination of a recurrent neural network (RNN) and a CNN to detect and track the myocardial landmarks through the image sequence for each slice. Radial and circumferential strain were then calculated from the motion of the landmarks and averaged on a slice basis.

**Results:**

Within the test set, myocardial end-systolic circumferential Green strain errors were −0.001 ± 0.025, −0.001 ± 0.021, and 0.004 ± 0.035 in the basal, mid-, and apical slices, respectively (mean ± standard deviation of differences between predicted and manual strain). The framework reproduced significant reductions in circumferential strain in participants with diabetes, hypertensive participants, and participants with a previous heart attack. Typical processing time was approximately 260 frames (approximately 13 slices) per second on a GPU with 12 GB RAM compared with 6–8 minutes per slice for the manual analysis.

**Conclusion:**

The fully automated combined RNN and CNN framework for analysis of myocardial strain enabled unbiased strain evaluation in a high-throughput workflow, with similar ability to distinguish impairment due to diabetes, hypertension, and previous heart attack.

Published under a CC BY 4.0 license.

*Supplemental material is available for this article.*

**Summary**

Fully automated whole-slice myocardial strain analysis is feasible in a high-throughput workflow by using a deep learning framework and can be used to detect impairment in disease groups (diabetes, hypertension, and previous heart attack) with confidence intervals similar to those attained with manual analysis.

**Key Points**

■ Test set myocardial end-systolic circumferential whole-slice Green strain errors (mean ± standard deviation of differences) were −0.001 ± 0.025, −0.001 ± 0.021, and 0.004 ± 0.035 in basal, mid-, and apical slices, respectively, when compared with manual ground truth; radial strain errors were −0.025 ± 0.104, −0.010 ± 0.100, and −0.009 ± 0.103, respectively.
■ This method enabled fully automatic strain analysis of tagged images, with no manual input required, at a speed of 13 slices per second on a computing processor with 12 GB RAM, as compared with 6–8 minutes per slice for manual analysis.
■ Significant reductions in circumferential strain found in the manual analysis for diabetes (95% confidence interval [CI]: 0.003, 0.020), high blood pressure (95% CI: 0.004, 0.010), and previous heart attack (95% CI: 0.008, 0.042) were reproduced in the automated analysis for diabetes (95% CI: 0.006, 0.019), high blood pressure (95% CI: 0.002, 0.008), and previous heart attack (95% CI: 0.009, 0.037).

## Introduction

Cardiovascular MRI tissue tagging is the noninvasive reference standard for myocardial strain estimation (1–4). Although cardiovascular MRI feature tracking allows calculation of strain from standard steady-state free precession images, features are limited to myocardial edges (5) and structures outside the myocardium (6), whereas cardiovascular MRI tagging enables detection and tracking of features within the myocardium. Displacement encoding with stimulated echoes (7,8) has the potential to provide higher spatial resolution strain estimates (9) but, to date, has not been as widely used (10). The utility of cardiovascular MRI tagging has been demonstrated in many different patient groups (4). However, there is a lack of robust fully automated analysis tools for the quantification of strain from cardiovascular MRI–tagged images, leading to analysis times that are prohibitive in a high-throughput setting, such as studies with many hundreds of cases or high-volume clinical centers with more than 20 cases per week (4,11,12).

The most common approaches for strain analysis of cardiovascular MRI–tagged images include profile matching and spline fitting (13), deformable contours (14), harmonic phase analysis (15), and sine wave modeling (16). However, these methods require manual initialization and lack robustness. Recently, deep learning methods, particularly convolutional neural networks (CNNs), have shown promise for general image processing, including automated cardiovascular MRI ventricular function analysis (17–20). However, there have been no reports using neural networks specifically designed for a robust analysis of myocardial motion and strain.

In this article, we developed a fully automated deep learning framework using two neural networks to estimate the left ventricular (LV) circumferential and radial strain on short-axis cardiovascular MRI–tagged images. The framework used spatial and temporal features to estimate the location and motion of myocardial landmarks, which were placed in consistent anatomic locations regardless of the overlying tag locations. Spatial features were extracted and learned using CNN architecture, while the temporal behavior was learned using a recurrent neural network (RNN) (21). This spatiotemporal neural network architecture was developed and validated on large-scale U.K. Biobank data (22) and, to our knowledge, it is the first to fully automatically estimate strains from cardiovascular MRI–tagged images in a high-throughput setting.

## Materials and Methods

### Data Set

This study examined 5065 U.K. Biobank participants who underwent cardiovascular MRI as part of the pilot phase (April 2014–August 2015) of the U.K. Biobank imaging enhancement substudy (22). A previous report described LV shape analysis in this cohort (23). Details of the image acquisition protocol have been described previously (22). The National Health Service National Research Ethics Service approved this study on June 17, 2011 (11/NW/0382). All participants gave written informed consent. An Aera 1.5-T (Siemens Healthineers, Erlangen, Germany) scanner running Syngo VD13A was used. Cardiovascular MRI–tagged images comprised gradient-recalled-echo images acquired in three short-axis slices (basal, mid, and apical) with the following parameters: repetition time msec/echo time msec, 8.2/3.9; flip angle, 12°; field of view, 350 × 241 mm; acquisition matrix, 256 × 174; voxel size, 1.4 × 1.4 × 8.0 mm; prospective triggering; tag grid spacing, 6 mm; temporal resolution, 41 msec; and approximately 20 reconstructed frames.

**Figure 1:**
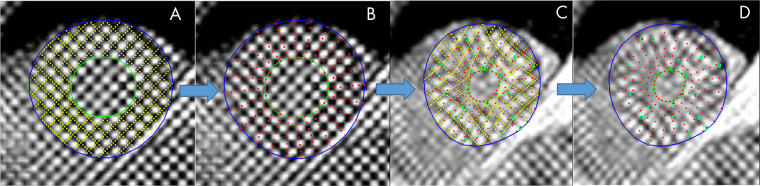
Manual myocardial landmark generation and tracking. *A* Placement of endocardial (green) and epicardial (blue) contours at end diastole (ED) (tag lines shown in yellow), *B* myocardial landmark points (red) at ED generated automatically, *C* tracked tag lines (yellow) at end systole (ES) with green points showing manual edits to the displacements, and *D* final landmarks (red) at ES.

Cases were distributed among five trained readers, and the images were analyzed with previously validated software (CIM, version 6.0; University of Auckland, Auckland, New Zealand) (9,24). Most cases (74%) were analyzed by two career image analysts (J.P., E.L.; 41% and 33% of cases, respectively), while the remaining cases were reviewed by three cardiologists (A.B., K.F., and E.M.; 12%, 9%, and 5%, respectively). The readers had 1–10 years of experience in cardiovascular image analysis. Each reader was trained according to a written standard operating procedure and satisfactorily completed at least 30 training cases before contributing toward the ground truth. Figure 1 illustrates the step-by-step process of generating the ground truth landmarks. The software identified and tracked 168 landmarks inside the myocardium at standard anatomic locations, beginning from the midpoint of the septum (halfway between the anterior and posterior right ventricular insertion points). The landmarks were equally spaced within the myocardium, with seven points in the radial (transmural) direction and 24 points in each circumference. The software used a deformable registration algorithm that attempted to track the tags by minimizing the sum of squared differences between consecutive frames (9,24). The readers manually corrected the tracking to match the motion of the image tags in several key frames: end diastole (ED, the first frame after detection of the R wave), end systole (ES, the frame of maximum contraction), after rapid filling, and at the end of the cycle. The software interpolated these corrections to the intermediate frames. Basal slices were not analyzed if the total circumference of the myocardium affected by the presence of LV outflow tract was 25% or greater; apical slices were not analyzed if there was no evidence of cavity at the ES. Cases also were excluded if the tagged image quality was deemed unacceptable by the readers. This resulted in 4508 cardiovascular MRI–tagging cases (12 409 slices), each with 168 landmark points tracked in each frame, that were available as ground truth for our neural networks. Participants with high blood pressure, diabetes, or previous heart attack were identified from the questionnaire data as having self-reported existing conditions, having conditions diagnosed by a physician, or taking medications for these conditions. Participant characteristics are shown in Table 1.

**Table 1:**
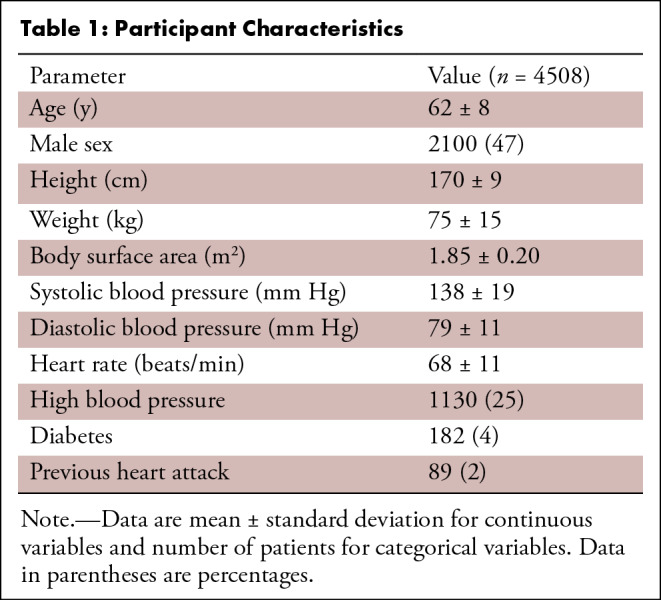
Participant Characteristics

### Framework Overview

**Figure 2:**
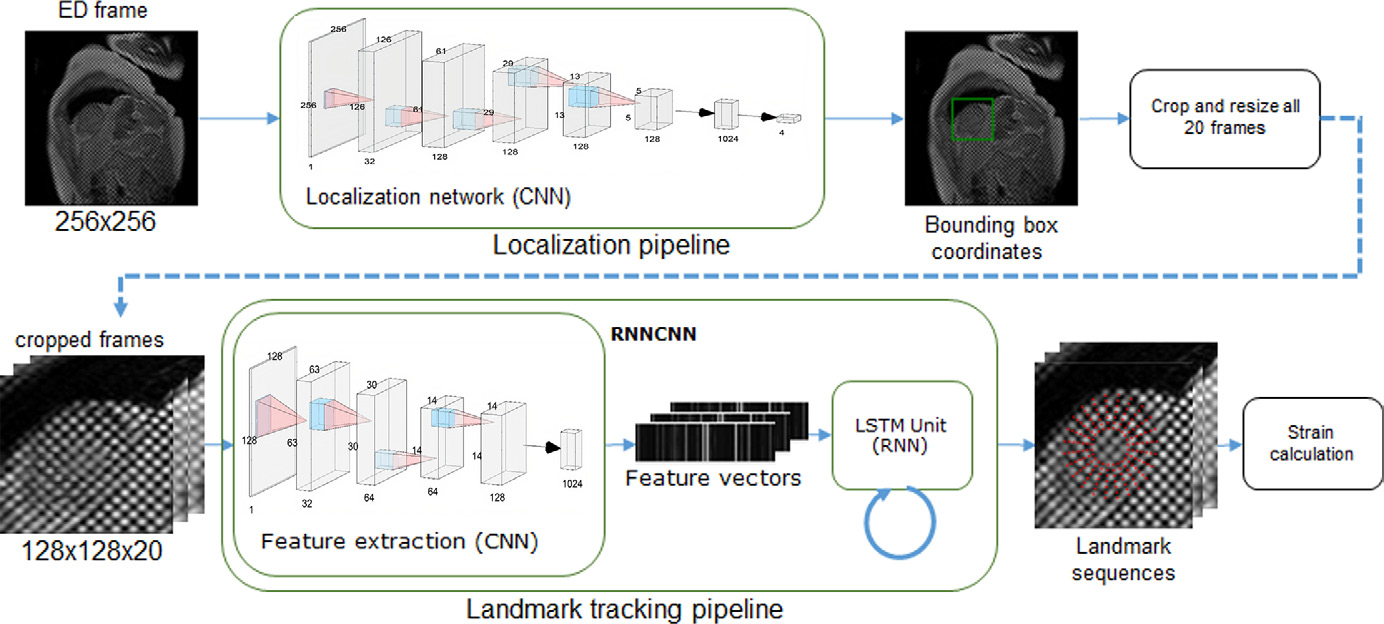
Overview of the machine learning framework for automatic myocardial strain estimation from cardiovascular MRI tagging. CNN = convolutional neural network, ED = end diastole, ES = end systole, LSTM = long short-term memory, RNN = recurrent neural network.

The deep learning framework consisted of the following steps: *(a)* find the region of interest (ROI) containing the LV myocardium, *(b)* crop and resample the image ROI for every frame, *(c)* detect and track myocardial landmarks across all the frames, and *(d)* calculate strains based on the motion of the landmarks (Fig 2).

Before images were input into the process, they were zero-padded to 256 × 256 matrix size, and each cine (sequence of frames in a slice) was fixed to 20 frames in length. Most slices (*n* = 12355) already had 20 frames; slices with fewer than 20 frames (*n* = 13) were padded with empty frames, and slices with more than 20 frames (*n* = 40) were truncated by taking the first 20 frames.

The neural networks were developed by using Tensorflow 1.5.0 (25) and Python and were trained on NVIDIA Tesla K40 (NVIDIA, Santa Clara, Calif) with 12 GB RAM. The final output of the framework were radial and circumferential strains, which were calculated from the displacement of landmark points for every time frame using the Green (Lagrangian) strain formula, which was compatible with finite strain tensors (9,26)
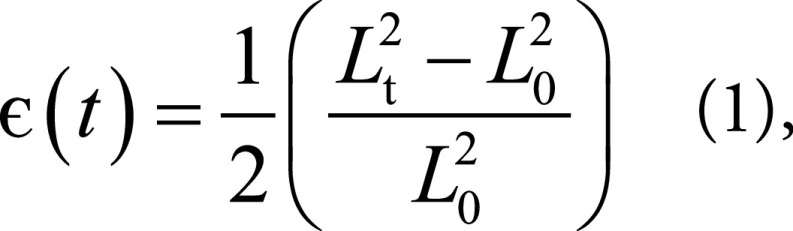
where *L*_t_ represents the segment length at any frame *t*, and *L*_0_ represents the initial length.

During training, the data were randomly divided, with 90% (*n* = 4056) of cases going into the training and validation set and 10% (*n* = 452) going into the test set. The first set was further partitioned, with 80% (*n* = 3244) of cases going into the training set and 20% (*n* = 812) going into the validation set, which was used for checking overfitting and convergence and tuning model parameters.

### ROI Localization

The localization network was designed to detect the ROI enclosing the LV myocardium in the ED frame. The output of this network was a rectangular bounding box defined by the extent of the myocardium, with a 60% increase to ensure enough spatial information was included from outside the myocardium. The network configuration is shown in Figure E1 (supplement). Each convolution layer was followed by batch normalization (27). A rectified linear unit (ReLU) was used as an activation function on every layer (28) except the output layer, which was a regression layer. A dropout layer (29) with 20% dropout probability was used after the first fully connected layer.

The network was optimized by using the mean squared error between the prediction and ground truth bounding box corners as the loss function. The Adam optimizer with a learning rate of 10^-3^ was used; learning rate was reduced by a factor of √2 for every fifth epoch after the 10th epoch. Accuracy was calculated by using the intersection over union (IoU) metric, defined to be the area of overlap of the predicted and ground truth bounding box, divided by the union of areas of the predicted and ground truth boxes.

After the ED frame bounding box was obtained, all the images in the cine clip were cropped using the same bounding box. Because at ED the heart is fully expanded before contraction, myocardium in the frames after ED has a smaller area, thereby ensuring the bounding box covers the myocardium in all frames. Subsequently, all the cropped images were resampled to 128 × 128 pixels by using bicubic interpolation to be fed into the landmark tracking network.

### Landmark Tracking

The landmark tracking network (combined RNN and CNN) was constructed from two components, a CNN component designed to extract the spatial features and an RNN component designed to incorporate the temporal relationship between frames. The input data for this network consisted of 20 frames (128 × 128 pixels) taken from the output of the localization pipeline. The combined RNN and CNN was trained end to end as a one network. Training time was approximately 10 hours.

A summary of the combined RNN and CNN architecture is shown in Figure E2 (supplement). A leaky ReLU (30) activation function (α = .1) was used in the shared-weight CNN component. The CNN component took one frame at a time and output a 1024-length feature vector per frame. The dynamic RNN (maximum of 20 frames) used a long short-term memory unit (31) with 1024 nodes. ReLU was used as an activation function in the RNN component. The final output layer was a regression layer, resulting in 168 landmark coordinates for 20 time frames.

The combined RNN and CNN was optimized using a composite loss function that simultaneously minimized position error and radial and midwall circumferential strain errors in each frame, defined on a slice-by-slice basis as follows (Fig 3):
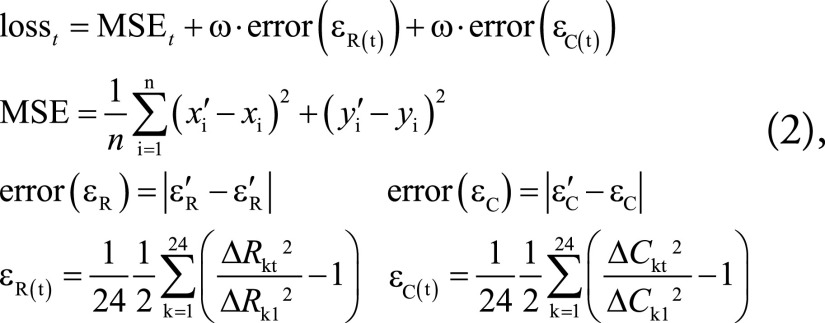
where *MSE*_*t*_ is the mean squared error between predicted (*x*_i_’*, y*_i_’) and ground truth (*x*_i_*, y*_i_) landmark positions at frame *t* (*n* = 168, the number of landmarks in one frame); Δ*R*_kt_ is the distance between the epi- and endocardial landmarks along each radial line *k* in frame *t*, with *t* = 1 being used as the reference frame; and Δ*C*_kt_ is the distance between two consecutive landmarks in the midwall circumference *k* at frame *t* (Fig 3). The strain errors were given weight (ω) to adjust for their relative scale compared with the displacement errors. The radial and circumferential strains (ε_R_ and ε_C_, respectively) were calculated using the Green strain formula (Eqq [1]). The strains were averaged over the slice before computing the error (Eqq [2]).

**Figure 3a:**
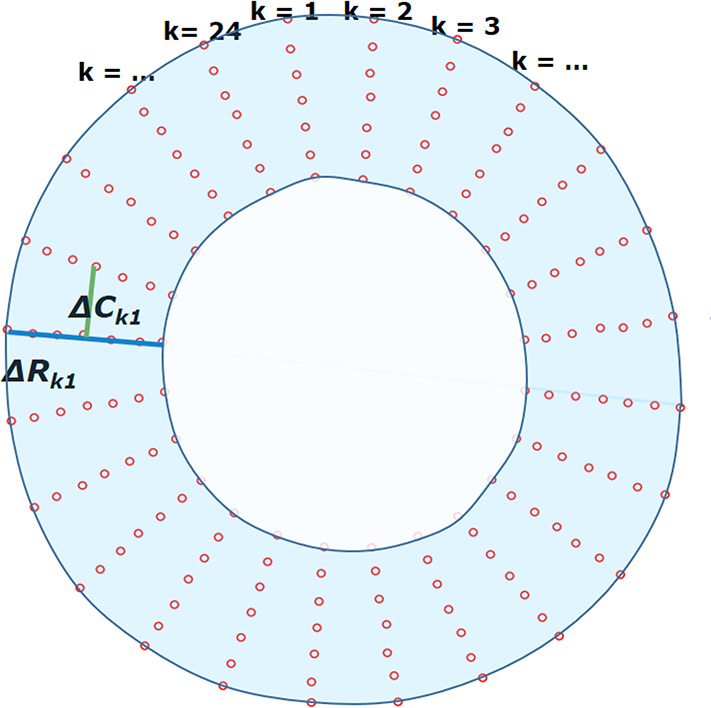
Measurements of radial and circumferential inter-landmark distances at **(a)** frame 1 (end diastole, assumed as the reference frame) and **(b)** frame *t*. Both images depict the seven circumferential rings of landmarks. Subendocardial, midwall, and subepicardial circumferential strain was calculated from the second, fourth, and sixth rings from the center, respectively. Δ*R*_kt_ shows the distance for radial line k in frame t, while Δ*C*_kt_ shows the distance for circumferential line *k* in frame t.

**Figure 3b:**
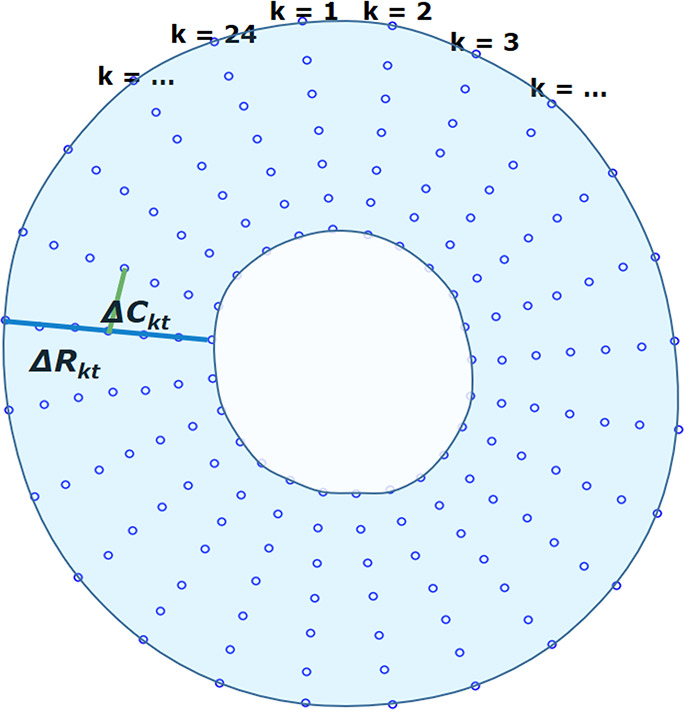
Measurements of radial and circumferential inter-landmark distances at **(a)** frame 1 (end diastole, assumed as the reference frame) and **(b)** frame *t*. Both images depict the seven circumferential rings of landmarks. Subendocardial, midwall, and subepicardial circumferential strain was calculated from the second, fourth, and sixth rings from the center, respectively. Δ*R*_kt_ shows the distance for radial line k in frame t, while Δ*C*_kt_ shows the distance for circumferential line *k* in frame t.

On the basis of the loss function, the network was effectively optimized using position (mean squared error) and strain (radial and circumferential) constraints. The Adam optimizer with a learning rate of 10^−4^ was used; the learning rate was reduced by a factor of √2 for every 10th epoch. Overall accuracy in the test set was calculated based on *(a)* slice-based strain errors at ES between the predicted strain and ground truth and *(b)* root mean squared position errors of all landmarks within a slice at ED and ES.

### Statistical Analysis

All statistical analyses were performed using SciPy Statistics (32), an open-source Python library for statistical functions. Continuous variables were expressed as mean ± standard deviation, with errors expressed as mean difference ± standard deviation of the differences computed over slices. We used the term *bias* to denote the mean difference and the term *precision* to denote the standard deviation of the differences. These were calculated across basal, mid-, and apical slices separately to give results for each location. Bland-Altman analysis was used to quantify agreement by plotting the difference against the mean of both measurements. Differences between automated and manual results, as well as interobserver differences, were assessed using a Student *t* test. A Bonferroni correction was used with 15 tests (Table 2), making *P* < .0033 indicative of a significant difference. Manual interobserver errors were obtained by comparing the landmark coordinates and strain differences (mean difference ± standard deviation of the differences, calculated over slices) between two observers for 40 cases. Differences in midventricular circumferential strain due to disease processes (diabetes, high blood pressure, and previous heart attack) were tested using a Welch unequal variances *t* test.

**Table 2:**
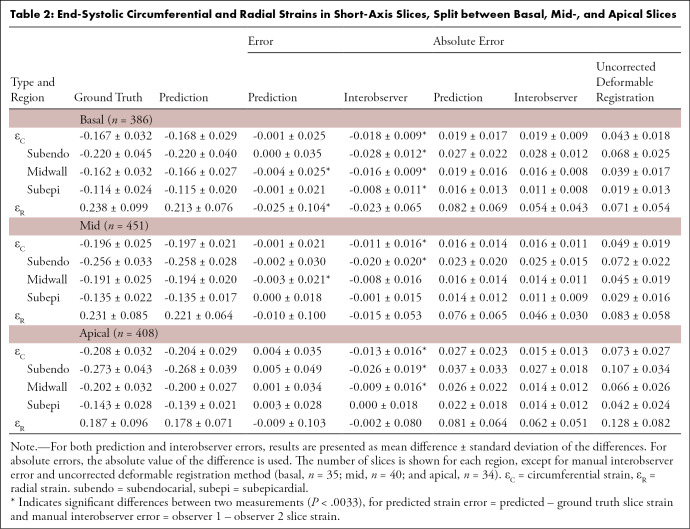
End-Systolic Circumferential and Radial Strains in Short-Axis Slices, Split between Basal, Mid-, and Apical Slices

## Results

### ROI Localization

The performance of the localization network was evaluated visually and quantitatively on the test data set (1245 slices). For visual evaluation, we reviewed the cases with the worst IoU and checked whether the cropped ROI was acceptable (ie, whether it contained the LV myocardium). None of the test cases were deemed to have an unacceptable ROI. The worst case had an IoU of 53%, and the average IoU for the test set was 90.4% ± 5.4. Figure 4 shows the worst and typical example results, as well as the accuracy distribution of the localization network.

**Figure 4a:**
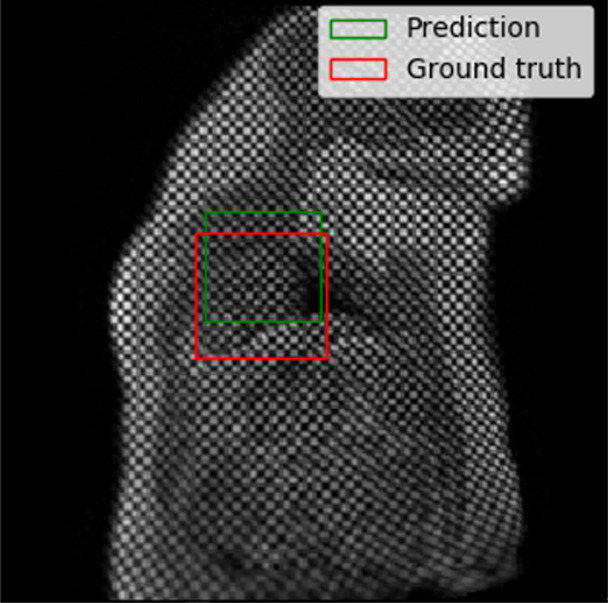
Example results from the localization network. **(a)** Intersection over union (IoU) of 53% and **(b)** IoU of 63% are the worst prediction results within the test set. **(c, d)** Typical prediction results. **(e)** Histogram shows the distribution of accuracy (IoU) throughout the test set.

**Figure 4b:**
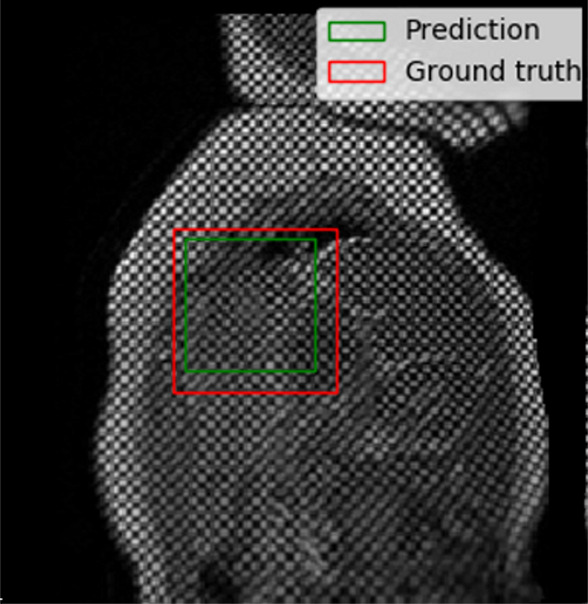
Example results from the localization network. **(a)** Intersection over union (IoU) of 53% and **(b)** IoU of 63% are the worst prediction results within the test set. **(c, d)** Typical prediction results. **(e)** Histogram shows the distribution of accuracy (IoU) throughout the test set.

**Figure 4c:**
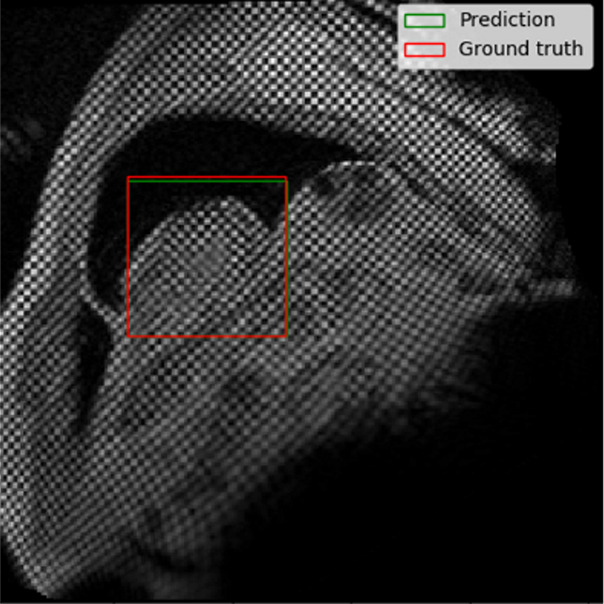
Example results from the localization network. **(a)** Intersection over union (IoU) of 53% and **(b)** IoU of 63% are the worst prediction results within the test set. **(c, d)** Typical prediction results. **(e)** Histogram shows the distribution of accuracy (IoU) throughout the test set.

**Figure 4d:**
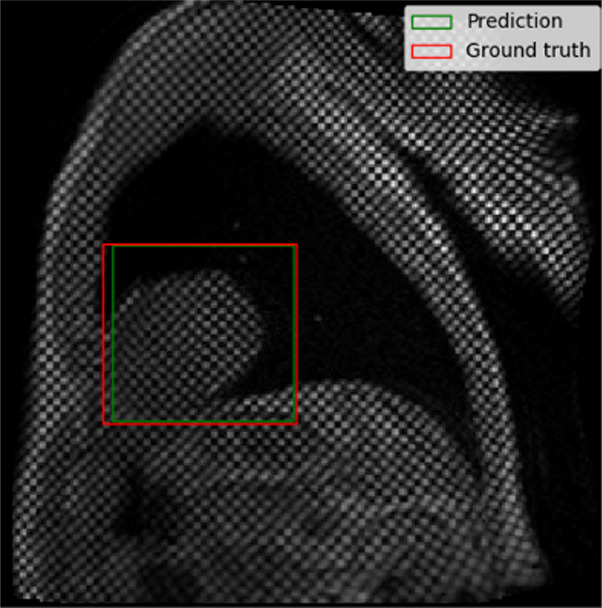
Example results from the localization network. **(a)** Intersection over union (IoU) of 53% and **(b)** IoU of 63% are the worst prediction results within the test set. **(c, d)** Typical prediction results. **(e)** Histogram shows the distribution of accuracy (IoU) throughout the test set.

**Figure 4e:**
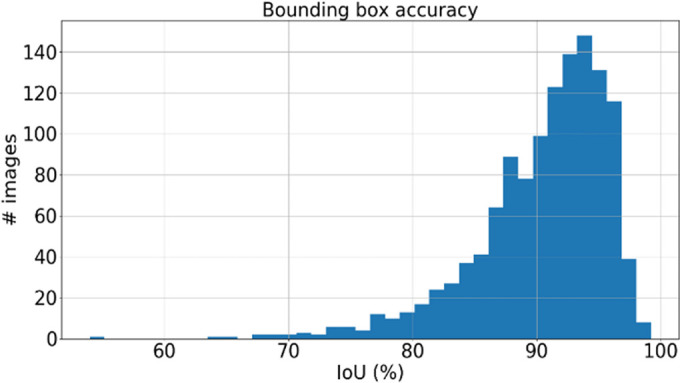
Example results from the localization network. **(a)** Intersection over union (IoU) of 53% and **(b)** IoU of 63% are the worst prediction results within the test set. **(c, d)** Typical prediction results. **(e)** Histogram shows the distribution of accuracy (IoU) throughout the test set.

### Landmark Tracking

The root mean squared position error of the 168 landmark coordinates on the test data set was 4.1 mm ± 2.0 at ED and 3.8 mm ± 1.7 at ES (mean over slices ± standard deviation over slices). In comparison, the interobserver position error on the 40 cases was 2.1 mm ± 1.8 and 2.0 mm ± 1.6 at ED and ES, respectively (mean ± standard deviation). Root mean squared position error was mainly due to variation in the placement of landmarks in the circumferential direction on the ED frame, corresponding to the localization of the midpoint of the septum from the right ventricular insertion points in the ground truth.

Visual checks were performed on the worst cases (IoU <70%, position error >4 mm, |error (ε_R_)| >0.2, or |error (ε_C_)| >0.05) as well as randomly selected cases to verify if the landmark positions were acceptable (ie, were located in the myocardium). Only one of 1245 slices (0.08%) had grossly misplaced landmarks, owing to the inaccurate prediction of the ROI (IoU = 53%), which caused the ground truth landmarks (16 of 168 in the ED frame) located outside the predicted ROI. Table 2 shows the predicted and ground truth strain values, with the mean and standard deviation of the differences between predicted and ground truth strains. In addition to the average strain over the whole myocardium, circumferential strain was also calculated for subepicardial, midwall, and subendocardial regions, using the second, fourth, and sixth circumferential ring of landmarks, respectively, as shown in Figure 3. All strain biases (mean difference) were small and not significantly different from zero, except for basal radial strain and basal and midventricle midwall circumferential strain (likely due to the relatively large number of image slices). The prediction precision (standard deviation of the differences) was worse than the interobserver precision, indicating that the observers were more precise than the network. As a further comparison, the absolute errors for the network and the absolute errors between the two observers are shown in Table 2. The interobserver absolute errors were similar to the network absolute error. Circumferential strains were highest for the subendocardial region and lowest for the subepicardial region, in agreement with previous studies (33). Subendocardial estimates showed better precision, with radial strains having the worst precision, which was in agreement with previous studies (9).

Indicative interobserver strain errors for two readers (*n* = 40) are also shown in Table 2 for comparison (similar interobserver differences were observed between the other readers). Small but significant differences in strain were mainly due to differences in contour placement. Similar interobserver differences using the same software in patients was found previously (9). The automatically predicted strain showed comparable errors to the manual interobserver strain errors, with worse precision but improved bias over a larger number of cases. As a further comparison, Table 2 also shows errors arising from the deformable registration method (using CIM, version 6.0, software) without manual correction on the 40 cases used to calculate the interobserver variabilities and with the average observer strain as the ground truth. These errors were larger than the automated RNN-CNN prediction.

**Figure 5:**
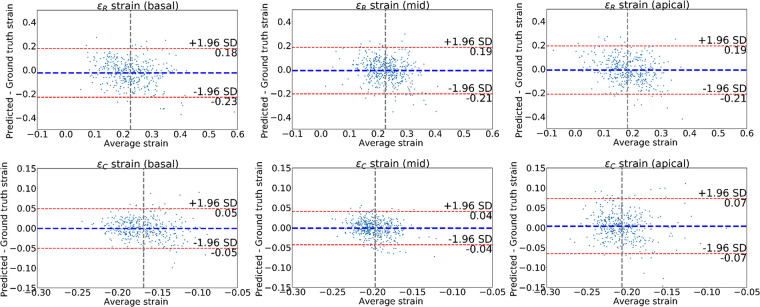
Bland-Altman plots of the strains obtained for the left ventricle at end systole. The strain values obtained from the predicted landmarks were compared with those from the ground truth landmark. The first row shows radial strains for three different short-axis slices; the second row shows average circumferential strains. Blue line denotes the mean difference; red lines denote the 95% limits of agreement (mean ± 1.96 · standard deviation [SD]).

Figure 5 shows Bland-Altman plots comparing the difference between the predicted and ground truth ES strains obtained from the landmarks. These allow us to confirm that the average of the differences for ε_R_ and ε_C_ was approximately zero. Most of the cases lie within the 95% limits of agreement (bias ± 1.96 · precision). Some outliers can be seen indicating a few cases with large error. The limits of agreement for ε_R_ were the widest, in agreement with previous studies that showed reduced accuracy for radial strain using cardiovascular MRI tagging (9). We observed the smallest limits of agreement for the ε_C_ on the middle slice and the largest limits of agreement for the apical slice.

**Figure 6:**
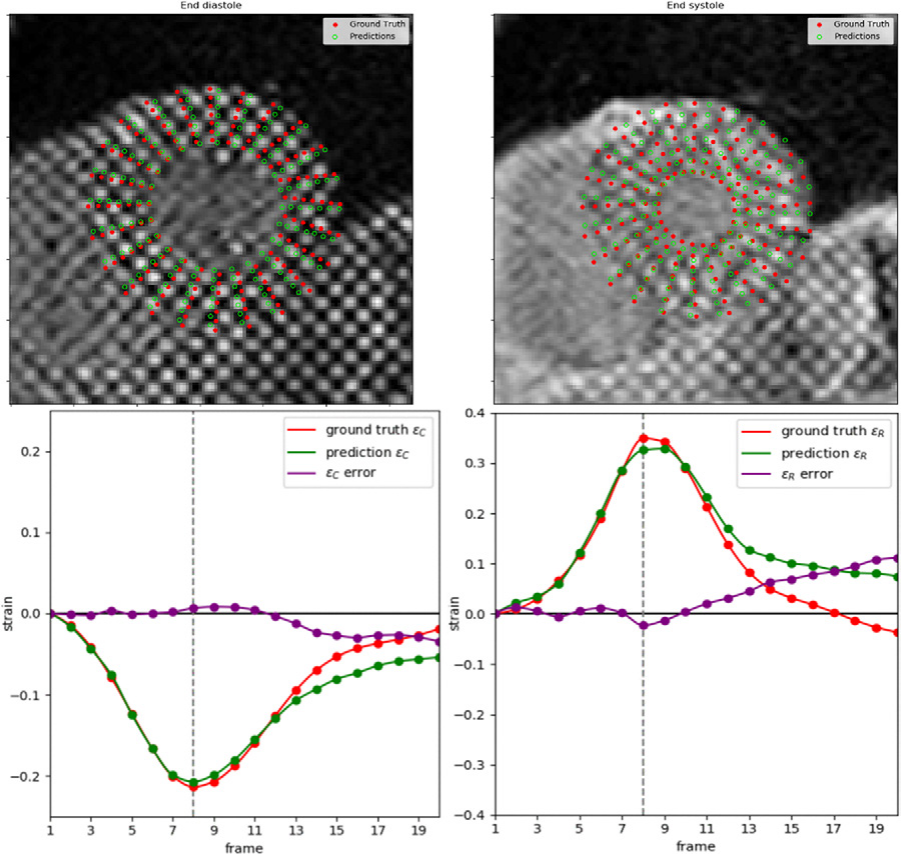
Example of ground truth compared with estimated landmarks during end diastole (ED) and end systole (ES) (top row) and strain calculation for the whole cine; circumferential strain (bottom left) and radial strain (bottom right). Vertical line marks the ES frame.

An example of the resulting landmark detection and tracking at ED and ES, including the strain estimations for all time frames, is shown in Figure 6. Tracking error tended to increase toward the end of the cine sequence when tag fading typically occurs during diastole. The fully automated framework could process images at approximately 260 frames (approximately 13 slices) per second with the computing processor. In comparison, manual analysis typically required 6–8 minutes per slice, yielding an approximately 5000-fold improvement.

Table 3 shows differences in midventricular circumferential strain for diabetes, high blood pressure, and previous heart attack, as self-reported by the U.K. Biobank participants. In each comparison, the manual analysis found statistically significant impairments between disease and reference (cases without hypertension, diabetes, or previous heart attack), which were reproduced with similar confidence intervals in each disease by the fully automatic method. LV mass and volume from MRI (34) are shown for comparison (indexed by body surface area). For high blood pressure, there were 818 cases in the training set, 195 cases in the validation set, and 108 cases in the test set. For diabetes, this was 135, 30, and 16 cases, respectively; for previous heart attack, this was 64, 13, and 11 cases, respectively.

**Table 3:**
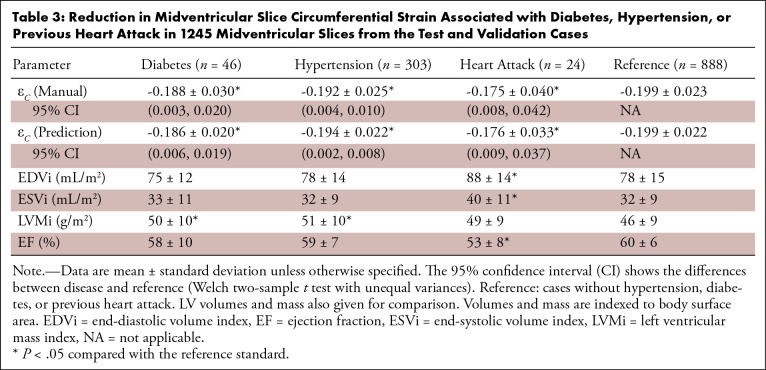
Reduction in Midventricular Slice Circumferential Strain Associated with Diabetes, Hypertension, or Previous Heart Attack in 1245 Midventricular Slices from the Test and Validation Cases

## Discussion

Strain estimation from cardiovascular MRI–tagged images is a challenging problem. We have designed a fully automated framework to calculate strains from cardiovascular MRI–tagged images and provide anatomic landmark points. To our knowledge, our study is the first to provide a fully automated analysis in a high-throughput setting. The method is feasible for direct application to the 100 000 participants in the U.K. Biobank imaging substudy, as these cases become available. The method was able to learn the previously validated deformable registration method and the manual correction of tracking errors. We are not aware of other fully automated methods that do not require some manual intervention for tagged cardiovascular MR images in practice. Our results suggest that the proposed framework can instantly (in real time) produce unbiased estimates of regional myocardial strains with reasonable precision, which reproduce differences due to disease processes. In a high-throughput clinical setting, this method can be used as a robust first-pass evaluation.

The performance of the fully automated framework is comparable with previous studies comparing cardiovascular MRI tagging with displacement encoding with stimulated echoes or feature tracking (9,11,35). In particular, a previous validation study using the same manually corrected tagging analysis procedure in patients (9) found interobserver circumferential strain errors of −0.006 ± 0.034 for tagging and similar errors between displacement encoding with stimulated echoes and tagging (9). Radial strains are known to be underestimated with tagging compared with displacement encoding with stimulated echoes and feature tracking (9) and have worse precision owing to the large tag spacing relative to the distance between the endo- and epicardium (3). In our study, both circumferential and radial strains at ES showed minimal bias. Although the basal ε_R_, basal midwall ε_C_, and midventricular midwall ε_C_ were statistically different (*P* < .0033), the magnitude of the bias (−0.025, −0.004, and 0.003, respectively) is unlikely to be clinically significant. As the network saw cases from all readers in the training set, it could learn an average of all readers and avoid the particular bias commonly associated with individual human readers.

Cardiovascular MRI tagging has also been incorporated into several large cohort studies, including the Multi-Ethnic Study of Atherosclerosis (36), and the U.K. Biobank cardiovascular MRI imaging extension (22). In particular, cardiovascular MRI tagging was included in the U.K. Biobank cardiovascular MRI protocol to provide accurate myocardial strain estimation for the analysis of developing disease (22). Fully automatic strain analysis would therefore improve the utility of cardiovascular MRI tagging in large cohort studies. This method can be applied for the automated analysis of the remaining U.K. Biobank cardiovascular MRI cohorts, which is estimated to be approximately 100 000 participants by 2023 and approximately 10 000 participants with repeat imaging 2–3 years after the initial imaging visit.

The root mean square position error was relatively high, owing to the difficulty in locating the precise midpoint of the septum. This was also seen in the interobserver position error. The error in position does not propagate to errors in strain, which depend only on the relative motions between ED and ES. However, strain errors are increased in the apical slice, due primarily to thin and obliquely oriented myocardial walls, partial voluming (different tissues averaged into a single voxel), and large motion artifacts (tags seem to disappear).

### Experiments and Hyperparameter Tuning

For the localization CNN, lower adjustment fraction (λ< 0.3) gave a more precise estimate of the LV ROI; however, it left the tracking network with reduced spatial context, which led to reduced accuracy. In future work, further augmentation, including arbitrary rotations and translation, might further improve accuracy. However, we found 90% IoU accuracy to be acceptable, and no cases failed outright (ie, not including the LV).

For the landmark tracking network, we also experimented with a separately trained landmark detection CNN and a subsequent landmark tracking RNN. The separately trained networks were inferior in performance, as the CNN can only process each frame independently and therefore did not guarantee motion coherence from frame to frame. The end-to-end combined RNN and CNN architecture was more difficult to optimize and sensitive to changes. Tweaking the network required a balance between the CNN and RNN components. Weights for the strain errors (ω = 1, 5, 10), batch size (20,25,30), CNN activation function (ReLU and leaky ReLU), number of long short-term memory nodes (400, 512, 600, 800, 1024, and 2048), and additional dense layers before and after the long short-term memory unit resulted in reduced performance during training. In the combined RNN and CNN network, we found that a leaky ReLU (37) was a key hyperparameter in the CNN component, which allowed negative values to be updated and prevented missing spatial information that might have been useful for the RNN component. The overall lean combined RNN and CNN architecture was inspired by Mari Flow (38).

### Limitations and Future Work

The neural networks used in this study were trained with one data set derived from the U.K. Biobank, which is homogeneous in the imaging protocol and consisted of mainly healthy participants (34). Additional augmentations to the data set are needed to adapt the neural network for a different data set, such as disease not seen in U.K. Biobank or data from different imaging protocols. Although the bias was excellent, more work is needed to improve the precision to match that of manual analysis. Table 2 shows that the network precision is approximately twice that of the interobserver error, which is adequate for large-scale studies such as U.K. Biobank but not for identifying subtle changes in individual patients. Another limitation was the number of frames, which is fixed (*n* = 20) owing to the nature of the current Tensorflow implementation. Future work will explore the calculation of segmental strains by assigning a segment label (according to the American Heart Association [39]) to every landmark. Additionally, other variants of RNN, such as bidirectional RNN (40) and convolutional long short-term memory (41), are possible candidates to improve the network by allowing a backward temporal relationship and preservation of spatial information, respectively. It would also be very useful to have an automatic evaluation of tag image quality, particularly in the context of tag fading, to determine when tags are not analyzable in part of the cardiac cycle.

### Code and Data Availability

This code is available online *(https://github.com/EdwardFerdian/mri-tagging-strain)*. In addition, the raw data, derived data, analysis, and results of this study are available from the U.K. Biobank central repository (application no. 2964). Researchers can request access to these data through the U.K. Biobank application procedure *(http://www.ukbiobank.ac.uk/register-apply/)*.

## Conclusion

In this study, we introduced a fully automated framework to estimate radial and regional circumferential strains from cardiovascular MRI–tagged images using a deep learning framework. The framework could detect and track 168 landmarks over many frames by using spatial and temporal features. The method resulted in unbiased estimates of reasonable precision suitable for robust evaluation in a high-throughput setting in which manual initialization or interaction is not possible. The method reproduced significant reductions in strain due to diabetes, hypertension, and previous heart attack.

## SUPPLEMENTAL FIGURES

Figure E1:

Figure E2:
