# Supplementary figures and images for: Fully Automated Myocardial Strain Estimation from Cardiovascular MRI–tagged Images Using a Deep Learning Framework in the UK Biobank
Source: Radiol Cardiothorac Imaging. 2020 Feb 27;2(1):e190032. doi: 10.1148/ryct.2020190032 (PMC7051160; doi:10.1148/ryct.2020190032)

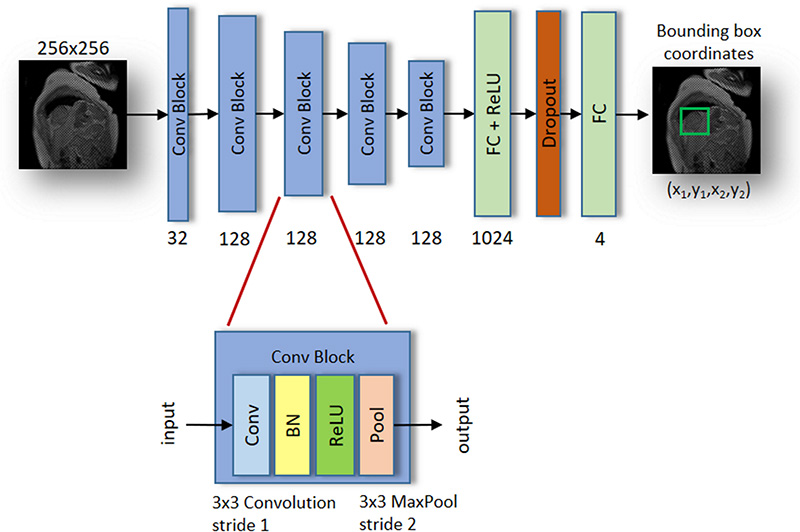

Supplement: Figure E1: [file ryct190032suppf1.jpg]
